# Influence of Breastfeeding in the Adaptation of and Absenteeism of Infants in Early Childhood Centers: A Preliminary Study

**DOI:** 10.3390/ijerph18020602

**Published:** 2021-01-12

**Authors:** Cristina Franco-Antonio, Esperanza Santano-Mogena, Sergio Cordovilla-Guardia

**Affiliations:** 1 Nursing Department, Nursing and Occupational Therapy College, University of Extremadura, Avda de la Universidad s/n, 10003 Cáceres, Spain; esantano@unex.es (E.S.-M.); cordovilla@unex.es (S.C.-G.); 2 Health and Care Research Group (GISyC), University of Extremadura, 10003 Cáceres, Spain

**Keywords:** breastfeeding, early childhood center, adaptation, absenteeism, infection

## Abstract

Schooling of children between 0 and 3 years old involves a process of adaptation and increases their exposure to infectious diseases, which leads to school absenteeism. Breastfeeding facilitates the development of secure attachment and protects the infant against infections. This study aimed to determine whether breastfeeding facilitates the adaptation of infants between 0 and 3 years old to early childhood center and decreases school absenteeism. A cross-sectional study was carried out by collecting data through a questionnaire, which was filled out by the parents and the childcare professionals. 160 infants participated. 40% of the infants who received infant formula from birth showed dependency behaviors (inconsolable crying or do not leave the caregiver for a long time) at the time of pick-up from the center, compared with 10%, 2.7%, and 2.6% of children breastfed between 0 and 6 months, and more than 6 and 12 months, respectively (*p* = 0.001). The interquartile range of absenteeism days per episode was 2–3 days for infants fed infant formula versus 1–2 days for those who were breastfed for more than 12 months (*p* = 0.041). Breastfeeding seems to be associated with fewer dependency behaviors at the time of collection and with fewer days of absence.

## 1. Introduction

In Spain, the schooling of children is not compulsory until 6 years of age [1]; however, the schooling of children between 3 and 6 years is currently almost at capacity, with rates exceeding 95% [2]. In addition, in recent years, there has been a significant increase in the enrolment of infants between 0 and 3 years old in early childhood centers-centers for the early care and primary education of infants-(ECE), with an increase in the school enrolment rate between 2002–2003 and 2015–2016 of 7.6% for infants under one year, 26.1% for one-year-olds and 35.1% for two-year-old, with rates in 2015-16 being 10.1%, 36.7%, and 57.2%, respectively [2,3].

The need for parents to place their infants in ECE centers has arisen in recent decades as a result of the incorporation of women into the labor market, thus establishing the need to create spaces for the care of infants to facilitate family reconciliation, as covered by European legislation, to encourage greater participation of women in the labor market [4]. Furthermore, in the case of Spain, where maternity leave ends in the sixteenth week postpartum [5], the need for this early schooling is urgent compared with that in other European countries with lengthier maternity and/or paternity leave.

For the infant population, the first year of attending ECE centers implies a new stage. For most of the infants, it is the first time they are separated from their family environment and exposed to society, introducing new social relationships in their lives, different from those maintained with their parents [6,7,8]. However, for parents, this stage also consists of a difficult challenge, as they feel insecure about how the infant will be treated, or guilty about the separation, which significantly influences the child’s expectations about the ECE center and can cause insecurity in the infant, causing that the adaptation and relationships with others to be more difficult and increasing their anxiety due to family separation [9].

The process of adapting a child to a school can be defined from two points of view: (i) the affective process, defined by Gervilla as “the way or process by which the child has to develop, from the point of view of their feelings, the loss and the gain that the separation implies, until arriving at an internal acceptance of themselves” [7], and (ii) the temporal process, defined by Lopez and Cantero as “the days, weeks or months that children take in reaching an adequate emotional, social and academic status in the early childhood center” [10].

Three stages usually appear in this process of adaptation [9], as follows: (i) the protest phase, where the children detach themselves from their attachment figure and feel threatened or in danger, with manifestations such as intense crying, tantrums, refusing food, tremors, fearful sleeping, vomiting, regressive behavior (e.g., thumb sucking, lack of sphincter control) and substitutive symptoms (night terrors, vomiting, tremors, refusing food); (ii) the ambivalence phase, in which they exhibit contradictory feelings, can cooperate with caregivers and partners and participate in activities, or they may present the symptoms of the protest phase at any given moment; and (iii) the adaptation phase, when the child can perform the school tasks as well as the rest of the classmates, according to their own abilities, they accept the caregiver, overcome anxiety and relate to other children.

Normally, adaptation to the center also has manifestations external to the center itself, such as the child agrees to go to the center, either happily or without protest; welcomes parents or relatives when they come to pick them up; and does not inappropriately increase their attachment behavior after having returned from the center [9].

The child’s ability to adapt to the ECE center will be influenced by the primary attachment relationships that the child has established since birth [9]. Establishing a secure attachment will help facilitate personal and social development. However, children with insecure attachments may exhibit problems of adaptation at this stage [9].

Attachment is the affective bond that is established as a result of the interaction between individuals who lead them to maintain proximity and contact to achieve security, comfort, and protection through the establishment of intense affective bonds [11]. After birth, the infant develops his/her main attachment relationship with his/her mother [12], as this maternal relationship will most often determine the establishment of a secure attachment [11]. This attachment becomes the basis of security from which the infant explores the world around them [9].

There is a series of behaviors in caregivers that facilitate the development of this type of attachment and encompass a series of thoughts and behaviors that adjust to the child’s signals and include recognizing them and attributing relevance to them, and they are related to the ability to manage the child’s anguish; express positive affection, empathy, and understanding; and have a range of affective responses according to the child’s ability to interact [13].

Some studies suggest that mothers who feed their infants with human milk have a better response to childhood crying and lower levels of abandonment [14,15,16]. This may be because, during breastfeeding (BF), a connection and dialogue are established between them [15]. In addition, at the neurochemical level, oxytocin and endorphins released during BF participate in the generation of attachment bonds, both in the mother and in the infant because both hormones are present in human milk [17,18]. Whether due to the direct influence of these factors or because BF could act as a marker of other positive behaviors related to the upbringing and the development of a secure attachment bond, BF could positively influence a better adaptation of the child to school [9].

On the other hand, early schooling has been associated with an increase in the incidence of infectious diseases, both respiratory and gastrointestinal [19,20,21,22], which translates into an increase in school absenteeism and the consumption of health resources [20].

Beginning BF in the first hour of life, exclusively BF during the first six months and continuing to supply human milk along with other healthy foods until at least two years of life, is considered the optimal feeding pattern for a child by the World Health Organization (WHO), the United Nations International Children’s Emergency Fund (UNICEF) and the Spanish Pediatric Association [23,24]. This dietary pattern influences the decrease in the risk of infection in the infants; thus, those infants fed with human milk have a lower risk of suffering lower respiratory tract infections and gastrointestinal infections and have a lower incidence of middle ear infection [25,26,27].

Therefore, the purpose of this study was to analyze whether there is an association between feeding at birth based on breastfeeding and the behavior associated with better or worse adaptation to the ECE center and illness-related absenteeism in infants enrolled in early childhood centers, under the hypothesis that breastfed infants adapt better to school and have lower rates of absenteeism associated with infectious pathologies.

## 2. Materials and Methods

A cross-sectional study was carried out. At all times, the voluntary and anonymous participation of the guardians of the minors and the ECE childcare workers was guaranteed, respecting at all times the ethical principles of the Helsinki’s Declaration [28]. The study was approved by the University Research Committee (20161130), and approval was requested from the Direction of the participating centers. Informed consent was requested from parents of participating infants and childcare professionals of the centers.

### 2.1. Setting

The study was carried out in four ECE centers (catering for infants from 0 to 3 years old) in Western Spain. In total, the four centers had 246 infants enrolled in the ECE centers. The data collection was carried out between February and March 2017. A total sample of 160 infants was obtained.

### 2.2. Measurement

The variables that were collected were:

1. Sociodemographic data, such as sex, age, number of siblings, duration of the child’s enrolment at the school, and who dropped off and picked up the infant from the center.
2. The type of feeding: the type of lactation at birth, the age at which BF was stopped if it had been initiated and the age at which supplementary feeding was introduced.
3. Adaptation of the infant to the center: To assess the adjustment of the infant to the center, the ECE childcare professionals were asked about negative behaviors [29] as (i) “wanting mother and father with him” during the childcare hours, (ii) “crying during the separation from the parents”, (iii) the frequency of crying during the childcare hours, as well as (iv) about the difficulties in the child’s interaction with their classmates. The parents were asked about negative behaviors [29] at the time of pick-up: (i) crying at the time of pick-up, (ii) showing dependency behaviors, as “not leaving the family member/caregiver who picked them up”, being aggressive or extremely sad, and (iii) about positive behavior [29] as (i) being happy at the time of pick-up, (ii) saying good-bye to the teacher and if they thought this behavior was normal. Likewise, they were asked for a personal assessment of the degree of adaptation of the infant to the school.
4. School absenteeism: The number of times the infant had been absent due to illness, average days of absence, and type of pathology that had caused the absences were assessed. Data were also collected on the existence of chronic disease or allergies that could interfere with the frequency of absenteeism of the child.

### 2.3. Data Collection

A questionnaire was developed that contained 14 questions to collect data on the children’s adaptation to the school, absences related to medical reasons, and the type of feeding that the child received since birth. This questionnaire consisted of questions addressed to the legal guardians of the children and the educators of the center responsible for their care.

To ensure accurate data collection, an informational meeting was held with the ECE childcare professionals of the centers to explain the objectives of the study and the use of the data collection questionnaires. Parents were asked to answer the adaptation questions by the parent/relative or caregiver who accompanied and picked up the child most of the time.

### 2.4. Data Analysis

For the statistical analyses, after verifying normality in the distribution of the quantitative variables, the median and interquartile range [IQR] were used as measures of central position and dispersion, applying, for the comparison of these variables, the Kruskal-Wallis test. To examine the association of categorical variables, Pearson’s chi-square and Fisher’s tests were used. A multivariate analysis was carried out using binary logistic regression for those variables with differences associated with the type of lactation. All analyses were performed using SPSS 24.0 for Windows (SPSS, Chicago, IL, USA), considering a significance value of *p* < 0.05.

## 3. Results

The 160 questionnaires collected corresponded to infants of a median age of 26 months (19–36). Once divided into groups according to the type of feeding received (Table 1), no significant differences were observed between them in terms of age, sex, number of siblings, or months of schooling in the center.

Fifteen (9.4%) minors were fed infant formula (IF) from birth, 70 (43.8%) received human milk between 0 and 6 months, 37 (23.1%) were breastfed more than 6 months but no more than 12 months, and 38 (23.7%) were breastfed for more than 12 months.

The age at which supplementary feeding was introduced (Table 1) was mainly between the sixth and seventh months for infants fed with human milk for more than 12 months; between the fifth and sixth months for infants fed formula from birth and those who received human milk for more than 6 months but less than a year, and between the fourth and sixth months for infants fed human milk between 0 and 6 months (*p* = 0.001) (Table 1).

With respect to the adaptation of the infants to the center (Table 2), we found that 6 (40%) of the infants who received IF at birth showed dependency behaviors at the moment of pick up from the center, compared with 7 (10%), 1 (2.7%), and 1 (2.6%) of the infants who were breastfed between 0 and 6 months, breastfed more than 6 months and breastfed more than 12 months, respectively (*p* = 0.001). In addition, adaptation was difficult for 6 (40%) of the infants fed from birth with IF, compared with 8 (11.4%), 7 (18.9%), and 5 (13.2%) infants fed with human milk between 0 and 6 months, more than 6 months, and more than 12 months, respectively (*p* = 0.062).

No differences were found regarding the existence of interaction problems of the infant during the stay in the center in relation to the food received.

In relation to school absenteeism (Table 3), 136 (85%) infants were occasionally absent from school due to illness since their schooling, with respiratory pathology being the main cause. The range of days of absence per episode was 2 to 3 days in children fed IF, 1 to 3 days for those breastfed between 0 and 6 months, 0 to 2 days for those breastfed for more than 6 months, and from 1 to 2 days for those breastfed for more than 12 months (*p* = 0.041).

The results of the multivariate analysis (Table 4) of the differences observed in relation to dependency behavior at pick-up show a protective effect of BF against this type of behavior. Thus, for infants breastfed for more than 12 months, between 6 and 12 months or between 0 and 6 months, compared with those fed with IF, the adjusted odds ratio (aOR) were 0.05 (CI 95%: 0.00–0.45), 0.05(CI 95%: 0.00–0.45) and 0.20 (CI95% 0.05–0.76), respectively, for this type of behavior.

## 4. Discussion

In our study, we found that BF was associated with a lower prevalence of dependency behaviors in the infants when they were picked up from school. The strong association of BF with this behavior could be a marker of better adaptation to the center, and the strength of the association increased with the duration of BF. However, it has also been found that breastfed infants are absent a fewer number of days per illness.

More than 90% of the infants belonging to our study population were breastfed at some point in their lives; however, in the first six months, cessation rates of BF were high, with the rate of infants still breastfed beyond the sixth month less than 50%. These statistics are similar to those obtained by other studies [27,30,31], which demonstrate the progress made in choosing BF as a method of infant feeding by the great majority of mothers but also demonstrate a high rate of BF abandonment in the first months of life.

Regarding the adaptation of the infant to the center, according to the assessment of the ECE childcare professionals, there were no differences found between the type of food received and crying episodes upon arrival at the center or during their stay, nor were there any interaction problems with other children or caregivers during the classroom activities. However, differences have been observed in terms of the overall adaptation of the infant when scored by the parents, who reported that the adjustment has been or is difficult for 40% of the infants fed IF. The association found between BF and a lower presence of dependency behavior by the infants at the time of pick-up at the center is consistent with that reported by some authors [14,15,16], which associates the practice of BF with the development of a bond, which is associated in turn with a lower presence of dependency behaviors in the absence of attachment figures [32]. In addition, these results are in accordance with the findings of Cornejo Dávila [33], who found that infants with a longer BF period were better at developing the skills that determine the autonomy of the child compared with infants who had not been breastfed. This protective effect of BF was not influenced by the age, sex, duration of schooling of the infant in the center, or absences due to illness.

Regarding the association between BF and absenteeism of children, in our study, large differences between established groups were not observed. Although infants who had been fed IF from birth had higher rates of absenteeism, 9 out of 10 of these infants had been absent due to illness since their schooling. However, we have found a difference in the number of days in which a child is absent per disease process, with a lower absenteeism rate among breastfed infants. This result could be related to the known protective effect of BF against the most common infectious processes, such as respiratory, gastrointestinal, and middle ear infection [25,26,27]. BF may not influence the number of episodes that the minor suffers because of the high exposure to all these types of processes due to early schooling, but it may facilitate a decreased intensity and faster resolution of disease [20,21,22].

However, differences have been found regarding the age at which supplementary feeding is introduced; specifically, such feeding occurs later in infants who have been breastfed for more than a year. This group was the only one in which the majority of infants did not start supplementary feeding before the sixth month, as recommended by the WHO [24] and supported by the latest systematic review [34], which did not find benefits in the earlier introduction of supplemental feeding but found an increased incidence of the absence of security associated with such earlier introduction.

### Limitations

The results obtained in this study, however, should be interpreted with caution due to the limitations inherent in this type of study. Due to the methodology used, it is not possible to affirm the causal nature of the relationships found. However, although we have tried to carry out the study at different centers, these results reflect the population studied and cannot necessarily be extrapolated to other populations or contexts. In addition, we must consider that some assessments of a child’s adaptation and behaviors may have observer bias; different parents may value the behavior of minors differently, where some behaviors may be normal for some and not for others. There may also have been recall bias, especially in the assessment of school absenteeism in minors. In addition, there may be a bias in the results due to the low representation of infants fed from birth with formula milk.

## 5. Conclusions

BF is associated with a lower presence of dependency behaviors at the time of pick-up of infants between 0 and 3 years of age who are enrolled in ECE centers, with the magnitude of the association being greater in infants breastfed for longer times. BF also appears to be associated with fewer days of absence due to the disease process, reducing absenteeism in infants enrolled in ECE centers.

Our findings support another argument to increase the efforts to generalize BF as an optimal way of feeding exclusively until the sixth month and supplementing it with a healthy diet until at least 2 years.

Given the results obtained and considering the design and sample of the study, future studies of greater scope and with a prospective cohort methodology are necessary, as such studies would allow data collection in and monitoring of infants from birth to three years of life. This would allow us to carry out more reliable causal inferences to measure the effect of BF on adaptation and absenteeism.

## Figures and Tables

**Table 1:** Characteristics of the children participating in the study.

	Total N = 160	IF N = 15	BF 0–6 Months N = 70	BF 6–12 Months N = 37	BF >12 Months N = 38	*p*
AGE (MONTHS)						
Median [IQR]	26 (19–31)	30 (24–35)	24 (18–30)	24 (13–31)	27 (21–33)	0.169 *
SEX N (%)						
Male	76 (47.5)	9 (60)	34 (48.6)	17 (45.9)	16 (42.1)	0.694 ^1^
Female	84 (52.5)	6 (40)	36 (51.4)	20 (54.1)	22 (57.9)	
Nº OF SIBLINGS						
Median [IQR]	0 (0–1)	0 (0–1)	0 (0–1)	0 (0–1)	0.5 (0–1)	0.681 *
TIME ENROLLMENT (MONTHS)						
Median [IQR]	8 (5–17)	17 (6–21)	7 (5–17)	10 (5–17)	6 (5–17)	0.260 *
SUPPLEMENTARY FEEDING (MONTHS)						
Median [IQR]	6 (5–6)	6 (5–6)	6 (4.5–6)	6 (5–6)	6 (6–7)	<0.001 *

* Kruskal-Wallis test; ^1^ Pearson Chi-square test; IQR: Interquartile range; BF: Breastfeeding; IF: Feeding by infant formula.

**Table 2:** Variables related to the child’s adaptation to schools.

n (%)	Total N = 160	IF N = 15	BF 0–6 Months N = 70	BF 6–12 Months N = 37	BF >12 Months N = 38	*p*
CRYING AT ARRIVAL						
All or almost every day	8 (5.0)	1(6.7)	3 (4.3)	1 (2.7)	3 (7.9)	0.629 ^1^
Occasionally or never	152 (95.0)	14 (93.3)	67 (95.7)	36 (97.3)	35 (92.1)	
CRYING IN THE CENTRE						
Every day	6 (3.8)	1 (6.7)	2 (2.9)	1 (2.7)	2 (5.3)	0.441 ^1^
On sporadic occasions	30 (18.8)	5 (33.3)	14 (20)	7 (18.9)	4 (10.5)	
Never or almost never	124 (77.5)	9 (60)	54 (77.1)	29 (78.4)	32 (84.2)	
PROBLEMS WITH INTERACTION						
Yes. frequently	12 (7.5)	0 (0.0)	6 (8.6)	5 (13.5)	1 (2.6)	0.455 ^1^
Only in some activities	25 (15.6)	4 (26.7)	10 (14.3)	4 (10.8)	7 (18.4)	
Never	123 (76.9)	11 (73.3)	54 (77.1)	28 (75.7)	30 (78.9)	
BEHAVIOR WHEN LEAVING THE CENTER						
Crying at pick up moment	2 (1.3)	1 (6.7)	0 (0.0)	0 (0.0)	1 (2.6)	0.097 ^1^
Dependency behaviors	15 (9.4)	6 (40)	7 (10)	1 (2.7)	1 (2.6)	0.001 ^1^
Normal behavior	138 (86.3)	14 (93.3)	60 (85.7)	32 (86.5)	32 (84.2)	0.921 ^2^
Aggressiveness	0 (0.0)	0 (0.0)	0 (0.0)	0 (0.0)	0 (0.0)	1.000 ^1^
Sadness	2 (1.3)	1 (6.7)	1 (1.4)	0 (0.0)	0 (0.0)	0.287 ^1^
ASSESSMENT OF ADAPTATION ACCORDING TO THE PARENTS’ POINT OF VIEW						
Very well	120 (75)	9 (60)	57 (81.4)	28 (75.7)	26 (68.4)	0.062 ^1^
Alright	26 (16.3)	6 (40)	8 (11.4)	7 (18.9)	5 (13.2)	
Difficult/Not adapted	14 (8.8)	0 (0.0)	5 (7.1)	2 (5.4)	7 (18.4)	

^1^ Fisher’s test; ^2^ Pearson Chi-square test. BF: Breastfeeding IF: Feeding by infant formula. Dependency behaviors: inconsolable crying; not living the person who pick-up them. Normal behavior: being happy, saying goodbye to the teacher.

**Table 3:** School absenteeism and its causes.

	Total N = 160	IF N = 15	BF 0–6 Months N = 70	BF 6–12 Months N = 37	BF >12 Months N = 38	*p*
CRONIC DISEASE n (%)	5 (3.1)	0 (0.0)	3 (4.3)	1 (2.7)	1 (2.6)	1 ^1^
ALERGIES n (%)	10 (6.3)	2 (13.3)	5 (7.1)	0 (0.0)	3 (7.9)	0.164 ^1^
ABSENTEEISM BY ILLNESS n (%)	136 (85.0)	14 (93.3)	61 (87.1)	27 (73.0)	34 (89.5)	0.116 ^2^
Nº OF ABSENCES Median [IQR]	4 (2–6)	5 (2–5)	3 (2–5)	2 (0–5)	2.5 (2–5)	0.154 *
Nº OF DAYS OF LACK PER PROCESS Median [IQR]	2 (2–3)	2 (2–3)	3 (1–3)	2 (0–2)	2 (1–2)	0.041 *
CAUSES OF ABSENCES n (%)						
Respiratory disease.	125 (80.6)	13 (86.13)	56 (82.4)	26 (74.3)	30 (81.1)	0.717 ^2^
Gastrointestinal disease	34 (21.9)	5 (33.3)	17 (25.0)	4 (11.4)	8 (21.6)	0.261 ^1^
Otitis media	27 (17.4)	4 (26.7)	13 (19.1)	5 (14.3)	5 (13.5)	0.635 ^1^
Other causes	14 (9.0)	0 (0.0)	6 (8.8)	4 (11.4)	4 (10.8)	0.652 ^1^

* Kruskal–Wallis test; ^1^ Fisher’s test; ^2^ Pearson Chi-square test. IQR: Interquartile range; INF: Infection; BF: Breastfeeding IF: Feeding by infant formula.

**Table 4:** Multivariate analysis. Dependence behaviors at the time of pick up from the center *.

Exposure	OR (CI 95%)	*p*
BF >12 months	0.05 (0.00–045)	0.008
BF >6 months	0.05 (0.00–0.45)	0.008
BF 0–6 months	0.20 (0.05–0.76)	0.018
IF	1.00 Ref.	
AGE		
1 month-increase	1.04 (0.96–1.13)	0.325
SEX		
Female	0.54 (0.16–1.80)	0.317
Male	1.00 Ref.	
SIBLINGS		
1 sibling	0.76 (0.27–2.14)	0.601
TIME ENROLMENT		
For each month of schooling	0.82 (0.72–0.94)	0.005
DAY OF ABSENCE PER EPISODE	0.76 (0.48–1.21)	0.241

* Binary logistic regression. Dependent variable: Ref. no dependency behavior. BF: Breastfeeding; IF: Feeding by infant formula; OR: Odds Ratio; CI: confidence interval. Ref: Reference.

## Data Availability

The datasets during the current study available from the corresponding author on reasonable request.
